# Development of Insecticide Resistance in Field Populations of Onion Thrips, *Thrips tabaci* (Thysanoptera: Thripidae)

**DOI:** 10.3390/insects14040376

**Published:** 2023-04-11

**Authors:** Waqas Wakil, Sehrish Gulzar, Shaohui Wu, Khawaja G. Rasool, Mureed Husain, Abdulrahman S. Aldawood, Michael D. Toews

**Affiliations:** 1 Department of Entomology, University of Agriculture, Faisalabad 38040, Pakistan; 2 Senckenberg German Entomological Institute, D-15374 Müncheberg, Germany; 3 Department of Entomology, University of Georgia, Tifton, GA 31793, USA; 4 Department of Plant Protection, College of Food and Agriculture Sciences, King Saud University, Riyadh 11451, Saudi Arabia

**Keywords:** *Thrips tabaci*, insecticide resistance, deltamethrin, lambda-cyhalothrin, imidacloprid, acetamiprid, spinosad, spinetoram, cypermethrin, abamectin

## Abstract

**Simple Summary:**

Onion thrips, *Thrips tabaci* Lindeman (Thysanoptera: Thripidae), are one of the most economically significant insect pests in onions, garlic, chives, leeks, and other *Allium* species. The resistance to different insecticides in thrips is a reality, but there is scarce literature available on this fact. The current study evaluates the status of insecticide resistance in eight geographically distinct field populations in comparison with a susceptible laboratory population of onion thrips in Pakistan using concentration–response bioassays on eight commonly used insecticides. Overall, all tested populations were found to have different levels of resistance varying with population location and chemicals. There were high levels of resistance noted in deltamethrin, mostly in populations from South Punjab, Pakistan. Among the insecticides, spinosyns remain effective and may provide better control of thrips in onion fields.

**Abstract:**

The present study evaluated insecticide resistance in field populations of onion thrips, *Thrips tabaci* Lindeman (Thysanoptera: Thripidae), collected from eight different onion-growing regions of Punjab, Pakistan. These field-collected populations were assessed for resistance development against eight commonly used active ingredients including deltamethrin, lambda-cyhalothrin, imidacloprid, acetamiprid, spinosad, spinetoram, cypermethrin, and abamectin. In leaf dip bioassays, *T. tabaci* adults showed varied levels of resistance towards different insecticides. Moderate or high levels of resistance to deltamethrin (58–86 fold), lambda-cyhalothrin (20–63 fold), and cypermethrin (22–54 fold) were observed in *T. tabaci* field populations. There were very low to moderate resistance levels to imidacloprid (10–38 fold), acetamiprid (5–29 fold), and abamectin (10–30 fold). The lowest levels of resistance were detected in thrips exposed to spinosad (3–13 fold) and spinetoram (3–8 fold). Insecticide resistance levels varied among populations collected from various geographic locations, but all populations exhibited elevated levels of resistance to deltamethrin. *Thrips tabaci* populations with higher resistance levels were most commonly found from the southern part of Punjab, Pakistan. Our findings revealed that spinosyns could be used as alternatives to conventional insecticides for the successful management of *T. tabaci* in onion fields.

## 1. Introduction

Onion thrips, *Thrips tabaci* Lindeman (Thysanoptera: Thripidae), are an economically important and polyphagous pest of onions, garlic, chives, leeks, and other *Allium* species throughout the world [1,2,3,4,5]. This pest has a distinct feeding behavior by puncturing and extracting cell contents from the leaf surface. It feeds in mesophyll cells, resulting in chlorophyll loss and ultimately decreased photosynthetic efficacy [6]. Feeding injuries caused by *T. tabaci* appear as silvery scars or patches on foliage [7]. Intense feeding on foliage provides an entry point for plant pathogens [8]. Heavy infestation due to *T. tabaci* can kill young plants [9], and severe injuries may reduce onion bulb yield [10]. Additionally, *T. tabaci* is a principal vector of iris yellow spot virus (IYSV), a widespread and severe disease in onions, irises, leeks, and other wild *Allium* spp. [11,12,13].

Although various pest control strategies have been recognized to manage thrips [14,15,16], control efforts often rely on the application of insecticides [17,18,19,20]. Unfortunately, thrips can be difficult to manage with insecticides because of their small body size and their cryptic and secluded behavior [9,21]. Hence, repeated insecticide applications are used to inhibit pest infestations. Lack of rotation partners and overreliance on insecticides can lead to the development of insecticide resistance in thrips populations [20]. Thrips populations can quickly develop insecticide resistance, mostly due to their short generations, parthenogenesis, and high reproduction rate [9,20,22,23,24].

The risk of resistance development has been demonstrated by extensive crop loss in control with organophosphates against *T. tabaci* since the 1990s [25]. In addition, there have been many reports on resistance development to pyrethroids in *T. tabaci* from the United States [26,27], New Zealand [2], Australia [28], and Canada [25]. Many other worldwide studies documented that onion thrips evolved resistance to synthetic pyrethroids (IRAC group 3A), organophosphates (IRAC group 1B), neonicotinoids (IRAC group 4A), and carbamates (IRAC group 1A) [2,25,27,28,29]. For example, previous resistance to deltamethrin [2,25,29], diazinon [2,25], lambda-cyhalothrin [25], dichlorovos [2,25], pyriproxyfen [30], spinosad [31], emamectin benzoate [31], and carbosulfan [31] in *T. tabaci* was observed from different regions of the world. However, there has been no report on insecticide resistance in onion thrips in Pakistan, and no documentation is available on the resistance of abamectin, cypermethrin, imidacloprid, acetamiprid, and spinetoram against onion thrips. The goal of this study is to evaluate the resistance to commonly used insecticides that belong to different insecticide classes, including pyrethroid (deltamethrin, lambda-cyhalothrin, and cypermethrin), neonicotinoid (imidacloprid and acetamiprid), spinosyn (spinosad and spinetoram), and avermectins (abamectin), in *T. tabaci* from different onion-growing areas in Punjab, Pakistan.

## 2. Materials and Methods

### 2.1. Test Populations

**Figure 1:**
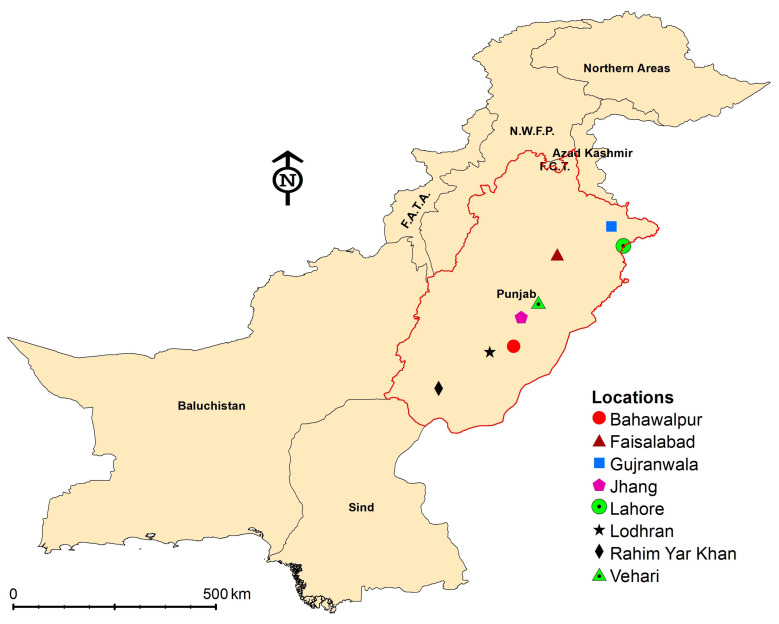
Geographical characteristics of eight different localities (Lodhran: 29°31′ N 71°37′ E; Jhang: 30°17′ N 72°19′ E; Faisalabad: 31°41′ N 73°07′ E; Gujranwala: 32°18′ N 74°19′ E; Vehari: 29°97′ N 72°42′ E; Rahim Yar Khan: 28°42′ N 70°29′ E; Lahore: 31°52′ N 74°35′ E; and Bahawalpur: 29°35′ N 71°69′ E) of onion fields in Punjab (Pakistan) where *Thrips tabaci* populations were collected for resistance bioassays.

Eight different field populations of *T. tabaci* were collected from onion plantations located in distant geographical areas of Punjab, Pakistan (Figure 1). All field populations of *T. tabaci* were collected in March, 2017. Heavily infested onion fields were selected for *T. tabaci* collection. These populations were used in laboratory bioassays directly after field collection. The susceptible population of *T. tabaci* was obtained from a stock colony maintained at the Microbial Control Laboratory, Department of Entomology, University of Agriculture Faisalabad, where the insect population was maintained for >2 years without exposure to any insecticides. The laboratory susceptible *T. tabaci* colony was reared by providing fresh cabbage leaves in large Petri dishes (150 mm in diameter). A small Petri dish (60 mm in diameter) was used as a water reservoir, and a cut on the side wall of this small plate was made for insertion of the cabbage leaf which, attached to the bottom of the large plate. A fresh cabbage leaf was placed onto dry filter paper at the bottom of the large plate, and the petiole of the leaf was inserted in the water reservoir, enclosed with a saturated cotton pad with distilled water, and covered with the plate lid. Fifteen to twenty adult thrips (female) were released onto the cabbage leaf in the large plate and covered with a lid. The large plate lid contained a fine sieve at the center of the plate for ventilation, and the lid was fastened to the bottom with two rubber bands. Then, the large plates were placed in an incubator at 25 °C and a 16:8 (L:D) h photoperiod. Water in the reservoir plate was refilled on a daily basis to maintain moisture. After 3–4 days, thrips were transferred onto new fresh cabbage leaves to maintain the thrips colony in the laboratory [32,33,34].

### 2.2. Test Chemicals

Detailed information on the tested insecticides is mentioned in Table 1. Pyrethroids and neonicotinoids were predominantly used by farmers with 2–4 applications in districts where *T. tabaci* populations were collected.

### 2.3. Bioassay

To evaluate the level of resistance among different populations of *T. tabaci* from various locations, the leaf dip method was used [23]. Bioassays were conducted against the adult life stage only. Insecticides were diluted with a 0.01% solution of Tween 80 (Merck, Kenilworth, NJ, USA), and six different concentrations were used for each insecticide (different concentrations were selected for each insecticide that produced >0% and <100% mortality in preliminary bioassays) in addition to an untreated control. Cabbage leaves used for the bioassay were collected from the horticulture area of the University of Agriculture, Faisalabad, and were not previously treated with chemical insecticides. Cabbage leaf discs (2.5 cm in diameter) were made using a cork borer and surface-sterilized with 70% ethanol solution for 2 min, followed by 2% sodium hypochlorite solution for 3 min and three subsequent rinses with distilled water. After surface sterilization, the cabbage leaf discs were dipped in different concentrations of insecticide solutions for 10s, and for the control group, it was dipped in a 0.01% solution of Tween 80 for the same period of time. After treatment, the leaf discs were allowed to dry for 1 h on a clean bench. Then, they were individually transferred to the center of small Petri dishes (60 mm) that were previously half filled with 2% water agar. Adults of *T. tabaci* (15 female adults) were transferred onto the leaf disc inside each Petri dish using a fine camel-hair brush. The Petri dishes were covered with lids that contained fine mesh for ventilation, and the plates were wrapped with parafilm to prevent thrips from escaping. The Petri dishes were incubated at 26 ± 2 °C and 60–70% relative humidity and a 13:11 (L:D) h photoperiod [35]. Each concentration per insecticide treatment was replicated six times. The mortality was recorded 48 h after treatment [23]. Thrips were considered dead if they were unable to move when disturbed with a fine camel-hair brush [23].

### 2.4. Statistical Analysis

The replicates were modeled as a completely randomized design. Treatment mortality was corrected for control mortality using the Abbott’s formula [36] and then subjected to probit analysis using Polo-Plus Software [37]. Lethal concentrations (LC_50_) along with their 95% fiducial limits were determined for each insecticide at each location. The resistance ratio of each insecticide at a specific location was determined by dividing the LC_50_ of a field population with the LC_50_ of the laboratory susceptible population. Significant differences in the susceptibility of the populations were established by nonoverlapping 95% confidence limits. The resistance ratios (RR) were categorized, according to the standard described by Ahmad and Arif [38], as no resistance (if RR < 1), very low (RR = 2–10), low (RR = 11–20), moderate (RR = 21–50), high (RR = 51–100), and very high (RR > 100).

## 3. Results

### 3.1. Deltamethrin Resistance

Varied levels of deltamethrin resistance were observed among different field populations of *T. tabaci*. All tested populations displayed high levels of resistance compared with the laboratory susceptible population. The highest resistance ratio (85.8 fold) was observed from the Jhang population, while the lowest (57.8 fold) was detected from the Lahore population (Table 2).

### 3.2. Lambda-Cyhalothrin Resistance

Varying with location, field populations of *T. tabaci* showed low to high levels of resistance to lambda-cyhalothrin. The population from Rahim Yar Khan exhibited the highest resistance ratio (63.4 fold), followed by Bahawalpur (56.7 fold); the rest of the populations showed moderate levels of resistance, but a low level of resistance (20.3 fold) was observed in Lahore. No significant differences were observed in populations from Lodhran (47.9 fold) and Jhang (49.8 fold) based on resistance ratio values (Table 3).

### 3.3. Imidacloprid Resistance

Very low to moderate levels of resistance were observed for imidacloprid against different populations of *T. tabaci*. The populations from Bahawalpur (38.0 fold), Vehari (31.2 fold), Rahim Yar Khan (27.1 fold), and Lodhran (24.0 fold) showed moderate levels of resistance. Only the population from Lahore (9.9 fold) showed a very low level of resistance. The populations from Jhang (19.3 fold), Faisalabad (14.7 fold), and Gujranwala (18.1 fold) showed low levels of resistance, while no significant differences were observed between Gujranwala and Jhang based on the resistance ratios (Table 4).

### 3.4. Acetamiprid Resistance

Very low to moderate levels of resistance to acetamiprid were observed among different field populations. The highest resistance was found in Jhang (29.3 fold), followed by Rahim Yar Khan (26.9 fold). Low levels of resistance were detected in Faisalabad (14.0 fold), Vehari (12.6 fold) and Bahawalpur (19.8 fold) populations. Very low levels of resistance were detected in Lahore (5.1 fold) and Gujranwala (10.1 fold) populations (Table 5).

### 3.5. Spinosad Resistance

Field populations of *T. tabaci* showed very low to low levels of resistance to spinosad. Only two populations, Lodhran (12.9 fold) and Rahim Yar Khan (11.3 fold), demonstrated low levels of resistance. The rest of the populations showed very low levels of resistance, with the lowest level of resistance observed in Gujranwala (2.6 fold) (Table 6).

### 3.6. Spinetoram Resistance

All tested populations of *T. tabaci* had very low levels of resistance to spinetoram. The level of resistance ranged from the lowest (2.7 fold) in Faisalabad to the highest (8.3 fold) in the Lodhran population (Table 7).

### 3.7. Abamectin Resistance

Populations of *T. tabaci* showed low to moderate levels of resistance to abamectin with the highest resistance observed in Vehari (30.4 fold), followed by Bahawalpur (28.8 fold). The lowest level of resistance to abamectin was detected in the Lahore population of *T. tabaci* (10.3 fold) (Table 8).

### 3.8. Cypermethrin Resistance

To cypermethrin, moderate to high levels of resistance were found among all populations of *T. tabaci*. Only the population from Jhang showed a high level of resistance (54.4 fold), while the rest of the populations had moderate levels of resistance. The minimum level of resistance (22.4 fold) was noted in the Lahore population of *T. tabaci* (Table 9).

## 4. Discussion

No published literature is available from Pakistan on the development of resistance in onion thrips to any of insecticides used in this study. In this study, we observed variable responses to deltamethrin in field-collected populations with a maximum level of 86 fold. Similar to the current findings, Maclntyre Allen et al. [25] observed the highest level of the resistance ratio in adults of *T. tabaci* ranging from 3.6–839 fold for deltamethrin in the population from onion fields in Ontario, Canada. Foster et al. [29] used 12.5 mg L^−1^ as a diagnostic dose/concentration of deltamethrin towards the laboratory susceptible population of *T. tabaci*, and they determined that the LC_50_ for field populations ranged from 350–1500 mg L^−1^ (28–120 fold). Moderate to high levels of resistance were observed in cotton whiteflies, *Bemisia tabaci* (Gennadius) (Hemiptera: Aleyrodidae), towards deltamethrin from the fields of Bahawalpur, Lodhran, Multan, Vehari, and Faisalabad districts of Punjab, Pakistan [39]. In the present study, other than deltamethrin, the pyrethroids lambda-cyhalothrin and cypermethrin were also found to have a stronger resistance than other insecticides, with 20–63- and 22–54-fold resistance levels, respectively. In 2011 and 2012, very low to high levels of resistance were observed in *Helicoverpa armigera* (Hübner) (Lepidoptera: Noctuidae) against cypermethrin from 15 different localities of the Punjab province [40]. However, Maclntyre Allen et al. [25] observed a relatively weaker resistance (2.0–13.1 fold) to lambda-cyhalothrin in thrips from onion fields in Ontario, Canada in 2001. Conversely, Herron et al. [28] revealed that the SA strain from Australia exhibited a maximum level of resistance of 606-fold to lambda-cyhalothrin. Shelton et al. [26] found very high levels of resistance to lambda-cyhalothrin in *T. tabaci* populations from New York, USA. In year 2000, Ahmad et al. [41] documented low levels of resistance to lambda-cyhalothrin in *B. tabaci* populations collected from Multan and its surroundings.

In the present study, *T. tabaci* developed neonicotinoid resistance, with a 10–38-fold increase in resistance to imidacloprid and 5–29-fold increase to acetamiprid. Similar to our findings, tobacco thrips, *Frankliniella fusca* (Hinds) (Thysanoptera: Thripidae), closely related to *T. tabaci*, were observed to have a 9.6-fold increase in resistance to imidacloprid in China, although no resistance (1–2 fold) to imidacloprid was observed until 2009, and with the passage of time, imidacloprid had lost its efficacy due to the development of resistance [23]. In the same study, a 1.02–8.75-fold increase in resistance was observed towards acetamiprid, which is similar to our results. Kahlid et al. [42] reported an 86-fold increase in resistance to imidacloprid and a 28-fold increase in resistance to acetamiprid from field populations of *B. tabaci* from the district Faisalabad, Pakistan, when exposed to the aforementioned insecticides up to five generations. In addition, according to the field study conducted in cotton fields by D’Ambrosio et al. [43], the neonicotinoids imidacloprid and abamectin were found ineffective to control larval populations of *F. fusca*. The current study detected low to moderate resistance to abamectin (10–30 fold), a similar level to the neonicotinoids in *T. tabaci* populations. Ahmad and Akhtar [44] documented very low to very high levels of resistance in *B. tabaci* toward the abamectin insecticide sampled from southern Punjab, Pakistan.

Spinosad and spinetoram are newly developed insecticides that have been widely used in thrips management systems. The current study exhibited low to very low resistance levels to spinosad and spinetoram against *T. tabaci*. Similar to our study, Wang et al. [23] observed that spinosad and spinetoram exhibited high levels of toxicity to *F. occidentalis* among all tested insecticides. Their results indicated that *F. occidentalis* populations in China were becoming less vulnerable to spinosyns. The enormous use of spinosyns has directed to development of resistance [18,45]. Fu et al. [46] revealed that *Thrips hawaiiensis,* when selected with spinetoram for 20 generations in the laboratory, showed a 103-fold increase in resistance to spinetoram relative to the laboratory population. Furthermore, in an earlier study, field populations of *T. hawaiiensis* exhibited a low resistance level (4.09 fold) to spinetoram [47], which supports to our findings. Farmers have testified control failures of spinosad for managing thrips throughout the world [48,49,50,51]. For example, repeated applications of spinosad in greenhouses have produced resistant populations of thrips in the USA [51,52], with comparable results reported in Australia [53]. Keeping in mind the abovementioned resistant complaints from different countries, we should avoid excessive applications of spinosad and spinetoram in onion production systems so that they maintain their efficacy under the field conditions.

In this study, stronger resistances to different insecticides were observed from the areas of southern Punjab, including Lodhran, Bahawalpur, Multan, Rahim Yar Khan, Vehari, and Jhang, that are considered key areas for cotton production along with onion crops. Cotton grown in Pakistan is attacked by a variety of insect pests from seedling to harvesting stage. Farmers use multiple insecticide applications to combat these insect pests, and most of them were found resistant to different groups of insecticides. *Thrips tabaci* is one of the key insect pests of cotton that also reduces cotton yield, and the pest remains active throughout the season on cotton (May to November) and onion crops (December to April). Heavy exposure to insecticides by *T. tabaci* on cotton crops might be the reason for the pests’ resistance to different chemical insecticides.

## 5. Conclusions

Our study concluded that the continuous overuse of insecticides has directed the development of resistance in *T. tabaci* populations in Pakistan. Levels of resistance varied with insecticides and field locations of *T. tabaci*. Among the insecticides tested, spinosyns had the lowest resistance and could continue to be used for managing thrips, but excessive applications should be avoided to prevent further selection for resistance. However, since laboratory bioassays cannot completely mimic all factors acting in field conditions [54], these results should always be compared with field trials for a more reliable evaluation of insecticide performance. To avoid control failures from *T. tabaci* resistance in onion fields, integrated resistance management (IRM) methods must be adopted, incorporating local monitoring data of pest populations and treatment thresholds. It is essential for farmers to rotate the remaining effective pesticides from different chemistries and modes of action to avoid building up or aggravating resistance problems. Additionally, to decrease the overall use of chemicals and maintain the efficacy of insecticides, alternative management strategies, such as biological control tactics and microbial biopesticides, should be applied for the integrated management of *T. tabaci*. By testing the resistance levels of *T. tabaci* populations to different insecticides, our findings provide essential information for the optimized control of the pest problem and preclude applications of ineffective chemicals, reducing resistance development, and environmental hazards. Future research will be directed to developing an integrated management program for the sustainable control of *T. tabaci*.

## Figures and Tables

**Table 1:** Active ingredients and trade names of insecticides used against *Thrips tabaci* in the laboratory.

Insecticide Class	Active Ingredient	Trade Name	Recommended Dose	Active Ingredient
Pyrethroid	Deltamethrin	Deltamethrin 2.5% emulsifiable concentrate (EC)	250 mL/ac	25 g L^−1^
	Lambda-cyhalothrin	Karate 2.5% emulsifiable concentrate (EC)	330 mL/ac	25 g L^−1^
	Cypermethrin	Arrivo 10% emulsifiable concentrate (EC)	250 mL/ac	100 g L^−1^
Spinosyn	Spinosad	Tracer 240 soluble concentrate (SC)	45 mL/ac	240 g L^−1^
	Spinetoram	Radiant 120 soluble concentrate (SC)	50 mL/ac	120 g L^−1^
Neonicotinoid	Imidacloprid	Imidacloprid 25% wettable powder (WP)	250 g/ac	250 g kg^−1^
	Acetamiprid	Acelan 20% soluble liquid (SL)	125 g/ac	200 g kg^−1^
Avermectins	Abamectin	Abamectin 1.8% emulsifiable concentrate (EC)	500 mL/ac	18 g L^−1^

**Table 2:** Lethal concentrations of deltamethrin to kill 50% of the tested population (LC_50_) of adult *Thrips tabaci* collected from eight geographical locations and a laboratory susceptible population (*n* = 540 for each population and each insecticide).

Population	Slope (SE)	LC_50_ (mg L^−1^)	95% Fiducial Limits	X^2^ (df = 4)	^a^ *p*	^b^ RR_50_
Lodhran	1.49 ± 0.24	734.67	781.14–1114.7	0.37	0.98	75.7
Jhang	1.39 ± 0.22	832.44	644.98–1298.70	2.34	0.67	85.8
Faisalabad	1.94 ± 0.37	615.38	474.11–988.24	0.46	0.97	63.4
Gujranwala	2.10 ± 0.42	634.38	477.73–1095.68	0.90	0.92	65.4
Vehari	1.47 ± 0.23	798.20	617.82–1248.53	3.79	0.43	82.3
Rahim Yar Khan	1.41 ± 0.21	678.86	546.87–966.42	0.57	0.91	70.0
Lahore	1.37 ± 0.19	561.08	467.23–738.72	0.57	0.96	57.8
Bahawalpur	1.59 ± 0.26	710.11	554.64–1091.23	0.51	0.97	73.2
Laboratory	0.65 ± 0.05	9.70	8.85–10.55	3.14	0.41	-

^a^ *p* = Goodness of fit test. ^b^ RR_50_ represents the resistance ratio = LC_50_ field population/LC_50_ susceptible population.

**Table 3:** Lethal concentrations of lambda-cyhalothrin to kill 50% of the tested population (LC_50_) of adult *Thrips tabaci* collected from eight geographical locations and a laboratory susceptible population (*n* = 540 for each population and each insecticide).

Population	Slope (SE)	LC_50_ (mg L^−1^)	95% Fiducial Limits	X^2^ (df = 4)	^a^ *p*	^b^ RR_50_
Lodhran	1.35 ± 0.19	313.95	257.80–427.48	0.62	0.96	47.9
Jhang	1.29 ± 0.18	326.81	268.67–444.42	0.93	0.92	49.8
Faisalabad	1.73 ± 0.28	179.04	148.30–223.62	0.08	0.99	27.3
Gujranwala	2.03 ± 0.38	218.84	174.84–306.68	4.72	0.31	33.4
Vehari	1.43 ± 0.20	262.44	219.07–342.32	3.44	0.48	40.0
Rahim Yar Khan	1.39 ± 0.22	416.22	322.49–649.38	2.34	0.67	63.4
Lahore	1.76 ± 0.29	133.49	107.07–161.84	0.24	0.99	20.3
Bahawalpur	1.59 ± 0.26	371.75	287.63–584.68	1.27	0.86	56.7
Laboratory	0.72 ± 0.05	6.56	5.91–7.20	4.02	0.40	-

^a^ *p* = Goodness of fit test. ^b^ RR_50_ represents the resistance ratio = LC_50_ field population/LC_50_ susceptible population.

**Table 4:** Lethal concentrations of imidacloprid to kill 50% of the tested population (LC_50_) of adult *Thrips tabaci* collected from eight geographical locations and a laboratory susceptible population (*n* = 540 for each population and each insecticide).

Population	Slope (SE)	LC_50_ (mg L^−1^)	95% Fiducial Limits	X^2^ (df = 4)	^a^ *p*	^b^ RR_50_
Lodhran	1.18 ± 0.15	113.62	96.54–143.75	3.25	0.51	24.0
Jhang	1.26 ± 0.16	91.47	78.81–111.27	0.81	0.93	19.3
Faisalabad	1.42 ± 0.19	69.33	59.29–82.46	1.93	0.74	14.7
Gujranwala	1.31 ± 0.17	85.72	73.81–103.75	0.81	0.93	18.1
Vehari	1.73 ± 0.30	147.34	112.30–241.92	2.00	0.73	31.2
Rahim Yar Khan	1.25 ± 0.17	128.33	106.29–171.66	1.81	0.76	27.1
Lahore	1.16 ± 0.13	46.87	40.13–53.49	18.94	0.11	9.9
Bahawalpur	1.34 ± 0.21	179.58	137.62–287.44	3.37	0.49	38.0
Laboratory	0.67 ± 0.05	4.73	4.31–5.14	4.10	0.39	-

^a^ *p* = Goodness of fit test. ^b^ RR_50_ represents the resistance ratio = LC_50_ field population/LC_50_ susceptible population.

**Table 5:** Lethal concentrations of acetamiprid to kill 50% of the tested population (LC_50_) of adult *Thrips tabaci* collected from eight geographical locations and a laboratory susceptible population (*n* = 540 for each population and each insecticide).

Population	Slope (SE)	LC_50_ (mg L^−1^)	95% Fiducial Limits	X^2^ (df = 4)	^a^ *p*	^b^ RR_50_
Lodhran	1.76 ± 0.34	72.34	48.59–168.69	0.96	0.91	20.8
Jhang	1.92 ± 0.42	102.07	60.15–371.60	0.21	0.99	29.3
Faisalabad	1.59 ± 0.27	48.64	36.92–80.50	0.22	0.99	14.0
Gujranwala	1.60 ± 0.25	34.98	28.24–49.38	3.75	0.44	10.1
Vehari	1.36 ± 0.20	43.94	35.09–63.87	1.17	0.88	12.6
Rahim Yar Khan	1.43 ± 0.28	93.46	50.40–241.53	2.69	0.61	26.9
Lahore	1.25 ± 0.15	17.80	15.41–20.48	7.54	0.11	5.1
Bahawalpur	1.39 ± 0.24	68.82	49.19–132.05	1.95	0.74	19.8
Laboratory	0.63 ± 0.04	3.48	3.18–3.78	2.81	0.22	-

^a^ *p* = Goodness of fit test. ^b^ RR_50_ represents the resistance ratio = LC_50_ field population/LC_50_ susceptible population.

**Table 6:** Lethal concentrations of spinosad to kill 50% of the tested population (LC_50_) of adult *Thrips tabaci* collected from eight geographical locations and a laboratory susceptible population (*n* = 540 for each population and each insecticide).

Population	Slope (SE)	LC_50_ (mg L^−1^)	95% Fiducial Limits	X^2^ (df = 4)	^a^ *p*	^b^ RR_50_
Lodhran	1.33 ± 0.20	3.86	3.06–5.72	0.85	0.93	12.9
Jhang	1.50 ± 0.21	1.58	1.33–1.87	0.55	0.96	5.3
Faisalabad	1.40 ± 0.18	1.15	0.95–1.34	1.07	0.89	3.8
Gujranwala	1.23 ± 0.14	0.79	0.62–0.93	6.73	0.15	2.6
Vehari	1.37 ± 0.20	3.09	2.54–4.21	1.31	0.85	10.3
Rahim Yar Khan	1.36 ± 0.20	3.40	2.75–4.79	1.65	0.79	11.3
Lahore	1.32 ± 0.17	0.82	0.64–0.98	6.03	0.19	2.7
Bahawalpur	1.55 ± 0.24	2.99	2.41–4.21	1.13	0.88	10.0
Laboratory	0.67 ± 0.05	0.30	0.25–0.35	1.72	0.02	-

^a^ *p* = Goodness of fit test. ^b^ RR_50_ represents the resistance ratio = LC_50_ field population/LC_50_ susceptible population.

**Table 7:** Lethal concentrations of spinetoram to kill 50% of the tested population (LC_50_) of adult *Thrips tabaci* collected from eight geographical locations and a laboratory susceptible population (*n* = 540 for each population and each insecticide).

Population	Slope (SE)	LC_50_ (mg L^−1^)	95% Fiducial Limits	X^2^ (df = 4)	^a^ *p*	^b^ RR_50_
Lodhran	1.66 ± 0.26	1.08	0.90–1.40	0.72	0.94	8.3
Jhang	1.50 ± 0.21	0.79	0.66–0.93	0.55	0.96	6.1
Faisalabad	1.11 ± 0.12	0.35	0.28–0.41	6.80	0.14	2.7
Gujranwala	1.33 ± 0.16	0.55	0.45–0.64	3.42	0.48	4.2
Vehari	1.41 ± 0.19	0.97	0.83–1.17	2.80	0.59	7.5
Rahim Yar Khan	1.24 ± 0.14	0.41	0.33–0.48	4.29	0.36	3.2
Lahore	1.30 ± 0.16	0.76	0.65–0.88	8.07	0.08	5.8
Bahawalpur	1.28 ± 0.15	0.74	0.64–0.86	6.24	0.18	5.7
Laboratory	1.22 ± 0.09	0.13	0.11–0.15	2.67	0.61	-

^a^ *p* = Goodness of fit test. ^b^ RR_50_ represents the resistance ratio = LC_50_ field population/LC_50_ susceptible population.

**Table 8:** Lethal concentrations of abamectin to kill 50% of the tested population (LC_50_) of adult *Thrips tabaci* collected from eight geographical locations and a laboratory susceptible population (*n* = 540 for each population and each insecticide).

Population	Slope (SE)	LC_50_ (mg L^−1^)	95% Fiducial Limits	X^2^ (df = 4)	^a^ *p*	^b^ RR_50_
Lodhran	1.57 ± 0.26	124.28	94.20–205.85	0.53	0.97	22.7
Jhang	1.58 ± 0.25	93.27	74.65–134.17	1.09	0.89	17.0
Faisalabad	1.38 ± 0.19	72.06	61.05–90.73	3.31	0.50	13.1
Gujranwala	1.62 ± 0.26	88.97	71.47–127.22	0.88	0.92	16.2
Vehari	1.36 ± 0.23	166.56	120.56–309.19	1.94	0.74	30.4
Rahim Yar Khan	1.59 ± 0.26	107.64	83.86–166.42	0.63	0.95	19.6
Lahore	1.18 ± 0.14	56.26	49.31–65.40	3.44	0.48	10.3
Bahawalpur	1.67 ± 0.31	157.79	111.41–316.15	1.80	0.77	28.8
Laboratory	0.63 ± 0.04	5.48	4.98–5.96	2.73	0.06	-

^a^ *p* = Goodness of fit test. ^b^ RR_50_ represents the resistance ratio = LC_50_ field population/LC_50_ susceptible population.

**Table 9:** Lethal concentrations of cypermethrin to kill 50% of the tested population (LC_50_) of adult *Thrips tabaci* collected from eight geographical locations and a laboratory susceptible population (*n* = 540 for each population and each insecticide).

Population	Slope (SE)	LC_50_ (mg L^−1^)	95% Fiducial Limits	X^2^ (df = 4)	^a^ *p*	^b^ RR_50_
Lodhran	1.47 ± 0.23	278.07	221.20–407.73	1.38	0.84	45.7
Jhang	1.57 ± 0.26	331.43	251.22–548.95	0.53	0.97	54.4
Faisalabad	1.84 ± 0.32	186.46	150.99–256.63	1.06	0.90	30.6
Gujranwala	1.61 ± 0.25	169.27	141.39–215.50	2.13	0.71	27.8
Vehari	1.50 ± 0.24	295.36	231.25–450.25	1.35	0.85	48.5
Rahim Yar Khan	1.87 ± 0.34	237.90	185.83–366.63	0.91	0.92	39.1
Lahore	1.61 ± 0.24	136.21	114.11–165.82	0.73	0.94	22.4
Bahawalpur	1.46 ± 0.22	253.55	205.06–357.23	1.49	0.82	41.6
Laboratory	0.66 ± 0.05	6.09	5.50–6.66	3.34	0.11	-

^a^ *p* = Goodness of fit test. ^b^ RR_50_ represents the resistance ratio = LC_50_ field population/LC_50_ susceptible population.

## Data Availability

Data is contained within the article.
